# Ocular metastases profile in a tertiary hospital in São Paulo, Brazil

**DOI:** 10.1186/s40942-024-00551-7

**Published:** 2024-04-16

**Authors:** Matheus Senna Pereira Ogata, Guilherme Rodrigues Ferreira, Melina Correia Morales, Arthur Gustavo Fernandes

**Affiliations:** 1 Department of Ophthalmology and Visual Sciences, Paulista Medical School, Federal University of São Paulo– UNIFESP, São Paulo, SP Brazil; 2 Department of Anthropology and Archaeology, University of Calgary, 2500 University Dr NW, T2N 1N4 Calgary, AB Canada

**Keywords:** Ocular oncology, Metastases, Tumours

## Abstract

**Objective:**

Ocular metastases are the most common intraocular tumours in adults. Data regarding the occurrence of these tumours in the Brazilian population is scarce. We aimed to investigate the profile of ocular metastases of patients referred to tertiary hospital service in São Paulo, Brazil.

**Design:**

Retrospective study.

**Participants:**

Patients referred to the Ocular Oncology service of the Federal University of São Paulo with initial diagnostic hypothesis of ocular metastasis.

**Methods:**

Data was retrospectively collected from medical records from June 2017 to June 2023. Age, sex, primary tumour site, previous knowledge of the systemic diagnosis, laterality, initial visual acuity (VA), local or systemic treatment and mean follow-up period were obtained.

**Results:**

A total of 37 cases were referred to the ocular oncology division due to a suspected ocular metastasis, 15 (40.5%) were confirmed. Mean age at diagnosis was 53.47 ± 16.01 years old, the majority (86.7%) of patients already knew the systemic diagnosis. Breast cancer (66.7%) was the most common primary site, followed by Lung cancer (26.7%). Both eyes were affected in 66.67% of the cases, all patients had metastases at the choroid (100.0%), and the mean initial VA was 1.37 ± 1.04 logMAR. Chemotherapy was the main systemic treatment modality (73.3%), and most patients had no ocular treatment (53.3%). The mortality rate along the follow-up period was 30.0%.

**Conclusions:**

Considering the number of new patients absorbed by the Ocular Oncology service over the study period, the frequency of ocular metastases was relatively low. The patients’ characteristics was comparable to data published in the international literature.

## Introduction

Ocular metastases are the most common intraocular tumors in adults [1] and might be one of the first signs of disseminated disease [2]. Cases of ocular metastasis are typically associated with a poor systemic prognosis [3]. Among the eye structures, the choroid responds as the most affected site, likely due to abundant blood flow within its tissues [3, 4]. Among women, breast cancer is the main primary tumor site for ocular metastases while among men the main primary site is lung tumor. Less frequent primary sites include prostate, gastrointestinal tract, and ovary [5].

Choroidal metastases typically present clinically as yellow masses, which may or may not be associated with serous detachment. Blurred vision, often reported as the primary complaint in metastatic patients, can be attributed to subretinal fluid accumulation. Multimodal analysis helps to differentiate metastases to other tumors, specially from amelanotic tumors such as hemangioma or choroidal melanoma [6]. Managing ocular metastases is not an easy task. There are many treatment modalities but no formal consensus to which course of action is the best in order not only to recover some visual function, but mainly to maintain a proper quality of life.

Data regarding ocular metastasis in Brazil are scarce, but a study conducted in the early 2000s has shown similar results to other American and European studies in terms of primary sites (i.e., lung and breast) and metastasis topography (i.e., choroid) [7]. In Brazil, the Public Health System (SUS - Sistema Único de Saúde) covers approximately 80% of the population [8]. It is organized into different levels of complexity; therefore, most cancer patients may need to be referred to a tertiary or quaternary center to receive adequate support over the course of their disease. However, many tertiary hospitals may lack specialized ophthalmology services to evaluate oncologic patients suspected of having metastasis.

The purpose of the current study was to investigate the profile of ocular metastases of patients referred to the Ocular Oncology Division at the Federal University of São Paulo, considering primary tumor site, laterality, ocular site, visual acuity, and proposed treatments.

## Materials and methods

Case records of patients who were referred to the Ocular Oncology service of the Federal University of São Paulo with an initial diagnostic hypothesis of ocular metastasis in the period of June 2017 to June 2023 were retrospectively included in the study. All suspected patients underwent a comprehensive ophthalmological examination, supplemented by multimodal assessments including fundus photography, fluorescein angiography (FA), optical coherence tomography (OCT), and ocular ultrasound. If compatible findings were identified in these examinations, a presumptive diagnosis was established, and the patient commenced follow-up care in our service. No patients underwent choroidal biopsy due to various reasons, including the inherent risks associated with the procedure, the high cost of vitrectomy, and the clinical conditions of the patients.

The study had approval from the UNIFESP Ethics Committee and was carried out in accordance with the tenets of the Declaration of Helsinki.

We evaluated the frequency of diagnosis confirmation as well as data on age, sex, laterality, primary tumor, previous knowledge of the diagnosis, initial visual acuity in LogMAR (VA), ocular and systemic treatments, follow-up time, and death occurrence for the confirmed cases.

Data were analyzed using the STATA 14.0 software (StataCorp LP, College Station, TX, USA). Frequency tables were used for descriptive analyses.

## Results

**Fig. 1:**
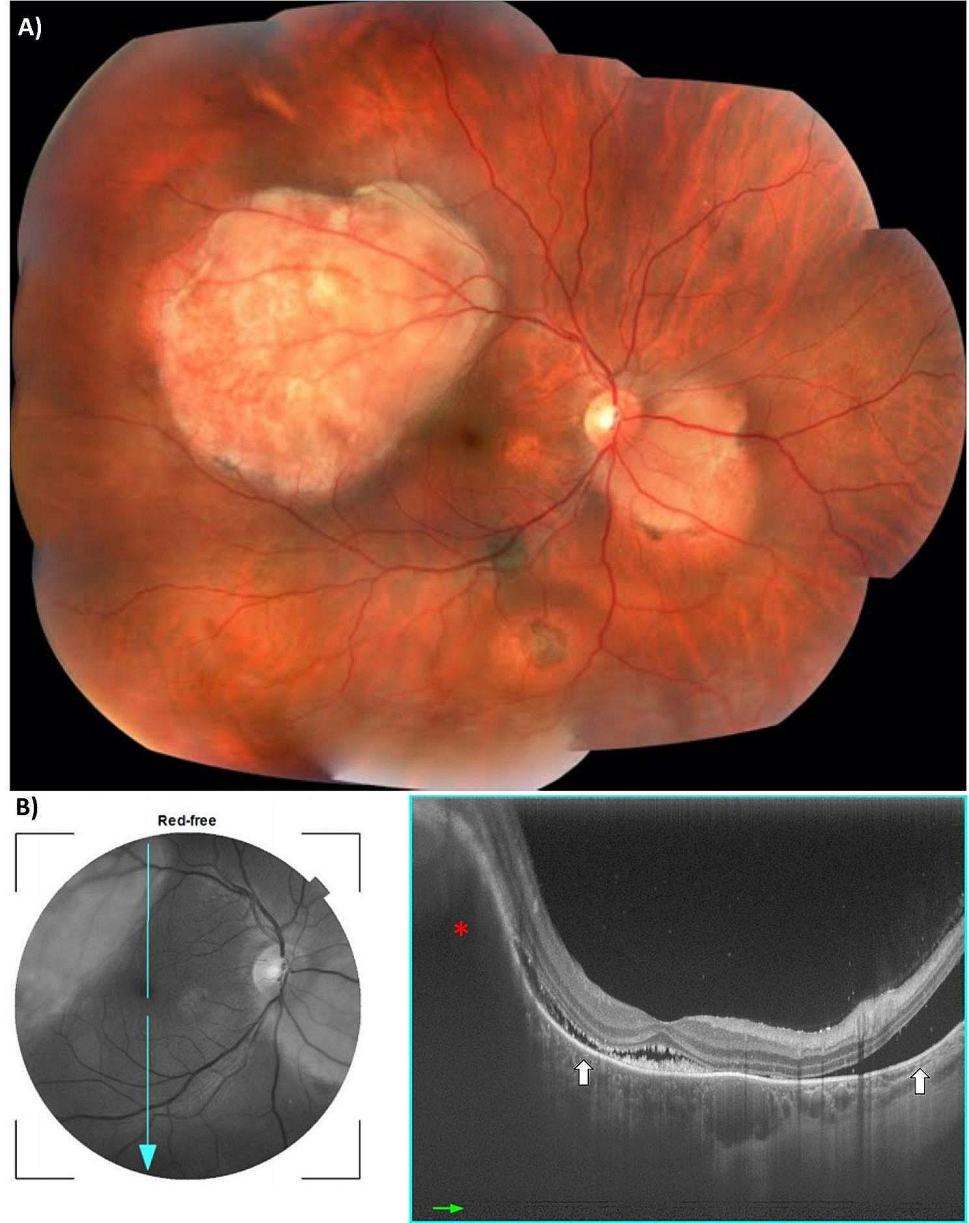
Image exams, right eye, male patient, 32 years old, case of ocular metastases from lung cancer primary site. (**A**) Fundus photography showing two pale yellow lesions: a larger one in the posterior pole (superior temporal quadrant) and the smaller one adjacent to the optic disc. (**B**) Red-free photography and Optical coherence tomography with red asterisk indicating the elevation of both the retina and choroid due to the presence of metastasis; white arrows are pointing the presence of subretinal fluid in the macula; hyperreflective material is shown in the subfoveal region

A total of 37 cases were referred to the ocular oncology division due to a suspected metastasis diagnosis. From those patients, 15 were confirmed (40.54%). Figure 1 shows image exams of one of our cases of confirmed ocular metastasis.

Along the study period, the Ocular Oncology division has received a total of 1,353 new patients referred with diagnosis hypothesis of ocular cancer, so that metastases corresponded for 1.11% of the cases in our service. Table 1 summarizes the characteristics of the 15 cases included in the analysis:

**Table 1:** Demographic and clinical characteristics of patients with confirmed metastasis diagnosis

**Sex***N*(%) Male Female	4 (26.67) 11 (73.33)
**Age ** *mean ± std*	53.47 ± 16.01
**Known systemic diagnosis** *N(%)* Yes No	13 (86.67) 2 (13.33)
**Primary site** *N(%)* Lung Mama Testicles	4 (26.67) 10 (66.67) 1 (6.67)
**Laterality** *N(%)* AO OD OS	10 (66.67) 4 (26.67) 1 (6.67)
**Ocular metastasis site** *N(%)* Choroid	15 (100.00)
**Visual acuity LogMAR** *mean ± std*	1.37 ± 1.04

There was a higher frequency of cases in women (73.3%) and most patients (86.7%) already knew the systemic diagnosis prior arriving at the ocular service. Breast cancer was the most common primary site (66.7%), followed by lung cancer (26.7%). All cases of breast cancer were females (100%) and most cases of lung cancer were males (75%). Both eyes were affected in 66.7% of the cases and all patients had metastases at the choroid. Mean visual acuity at diagnosis was 1.37 logMAR, corresponding to 20/468 on Snellen scale. All patients with previous knowledge of cancer had systemic metastatic disease concomitantly to ocular metastasis.

Regarding metastases treatment, most patients were treated with systemic chemotherapy. Table 2 shows the different options considered.

**Table 2:** Treatment characteristics of patients with confirmed metastasis diagnosis

**Systemic treatment***N*(%) Chemotherapy alone Chemotherapy + Radiotherapy Anastrazol No systemic treatment	5 (33.33) 6 (40.00) 1 (6.67) 3 (20.00)
**Ocular treatment** *N(%)* Radiotherapy Anti-VEGF No ocular treatment	2 (13.33) 5 (33.33) 8 (53.33)
**Follow-up time in months ** *mean ± std*	14.77 ± 12.69
**Deaths** *N(%)* Yes No	3 (20.00) 12 (80.00)

## Discussion

Choroidal metastases are the most common intraocular tumors [1], however, at the Ocular Oncology Service of UNIFESP the observed frequency was relatively low. Probably reasons for this discrepancy are likely to be justified by the fact that patients are often asymptomatic and access to ocular oncology centers in the country may be difficult.

Most primary tumors were known before the ocular diagnosis, which is in accordance with the current literature [4, 9]. Our data, however, may be biased because, as a tertiary center, our service is part of the Hospital São Paulo complex and all oncologic patients with ocular complaints are referred directly to us. The primary sites were breast or lung cancer as shown in previous reports from different populations. All cases were affecting choroid. Iris and ciliary body metastases are extremely rare and none of them were identified in our study.

Choosing a systemic or local therapy for ocular metastases is a decision that must consider many variables. As most choroidal metastases are bilateral and occur concurrently to other sites [9], systemic chemotherapy is a considerable option. However, in unilateral cases, our study showed that performing radiotherapy is also viable, as indicated in the literature [10, 11]. External beam radiotherapy was the modality used to treat the patients in our study, specifically in patients with larger choroidal masses and refractory subretinal fluid. Plaque radiotherapy is not available in the Brazilian public health system. It must always be remembered that the main goal is to improve progression-free survival [12], weighting the options to provide the best quality of life as possible, so the option “not to treat” many times is overlooked.

Patients with oligometastatic disease might benefit from a local only approach. Intravitreal injections of antiangiogenics have shown promise to treat choroidal metastases. The choroid has a rich vascular network and so does its metastases. Therefore, blocking angiogenesis might help decrease the tumor mass and its consequential side effects. One of the most common complications of metastases is subretinal fluid, which may cause a deep impact in VA. In our study, patients with fluid in the macular region and loss of VA were referred to local treatment with Anti-VEGF injections. In the Brazilian Health System, Bevacizumab is more easily available than Ranibizumab or Aflibercept due to its lower cost [15]. In the study, only 1/3 of patients have received intravitreal treatment and have shown mixed outcomes regarding VA and fluid reabsorbation. While previous studies recommend the use of Anti-VEGF agents to improve visual acuity and regress choroidal metastasis, it is not clear whether local therapy alone would suffice to resolve the choroidal lesion or if it would be more effective as an adjuvant to systemic chemotherapy [16].

Life expectancy is hard to quantify in choroidal metastasis patients and mortality in these patients correlates with the presence of synchronous lesions in the liver or lungs [12–17]. The study followed the patients in an average of 14.8 months and the survival rates were considerably high in this period.

This is the first study characterizing ocular metastases in a Brazilian group of patients, however some limitations need to be pointed. The retrospective design and data collection from medical records are subject to information bias. The follow up time in the service was short. And finally, the number of cases was low, which rises the reflection on difficulties of access to an ocular oncology center. Still, this study provides a great panorama on tumor profile and on how ocular complications in cancer patients are dealt with in a tertiary hospital in the largest city of Brazil.

## Conclusion

Our findings described the profile of patients with confirmed diagnosis of metastases in Brazil presenting individuals and lesions characteristics. Most cases didn’t have any ocular treatment and the mortality rates were considerable low. Multicentric studies are recommended to better characterize this population.
